# PROTOCOL: Use of community participation interventions to improve child immunisation in low‐ and middle‐income countries: A systematic review and meta‐analysis

**DOI:** 10.1002/cl2.1119

**Published:** 2020-09-28

**Authors:** Monica Jain, Mark Engelbert, Marie Gaarder, Avantika Bagai, John Eyers

**Affiliations:** ^1^ International Initiative for Impact Evaluation (3ie) New Delhi India

## 1 BACKGROUND

### 1.1 The problem, condition or issue

Immunisation remains one of the most cost‐effective interventions to prevent and control life‐threatening infectious diseases. A study estimating the health and economic impact of routine immunisation for the period 2001–2020, found that introduction and/or increased coverage of vaccines are projected to avert over 14 million deaths, 350 million cases of illness, 8 million cases of long‐term disability and 700 million disability adjusted life years (DALYs) (Ozawa et al., 2017). Yet rates of routine vaccination of children in low‐ and middle‐income countries (LMICs) are strikingly low or stagnant, leading to a high disease burden and high infant and child mortality. In 2017, an estimated 19.9 million infants worldwide were not reached with routine immunisation services. Around 60% of these children live in 10 low‐and‐middle income countries, including Ethiopia, India, Nigeria and Pakistan (“Immunization coverage,” 2018). Furthermore, national averages on immunisation coverage often obscure the underlying disparities within countries, and as a result, inequalities in immunisation often go unobserved or are underreported (Restrepo‐Méndez et al., 2016). Therefore, there is an urgent need for interventions that improve immunisation of children in these countries.

A number of innovative approaches to this problem have arisen in recent years. These approaches include strategies and technologies that enhance communities' participation in the planning, delivery, monitoring and uptake of routine vaccinations of children. These strategies have received considerable attention from funders, researchers, and practitioners (UNICEF, n.d.; WHO, 2008). However, there is at present a dearth of rigorous and systematic evidence about the effectiveness and cost‐effectiveness of these interventions. There is therefore a need to make such evidence available to guide policymakers and public health practitioners in making informed decisions about these interventions. This review will systematically analyse the literature on this topic, conduct causal chain analysis to identify key facilitators and inhibitors of intervention success, and perform cost‐effectiveness analysis to assess the value‐for‐money of enhancing community participation to improve immunisation of children.

### 1.2 The intervention

The review will focus on all interventions that use community participation or engagement to improve routine immunisation coverage of children.

#### 1.2.1 How do we define a community?

Communities may be identified in multiple ways. Writing in the *Bulletin of the World Health Organization*, Marston et al. (2016, p. 376) define communities as “groups of people who share common interests, concerns or identities in settings that are defined by geography, culture, administrative boundaries or geopolitical region or that are identified with joint activities, such as work or recreation.” In this systematic review (SR), we define “communities” in reference to the administrative boundaries of the lowest level of the health service delivery system (or whatever level provides routine immunisation services in the local context). We prefer this over those defined by a geographical boundary, such as a village, as in some contexts the lowest level of health service delivery system provide services to multiple villages. For the purposes of our review, a community comprises a group of people who are served by a particular primary health facility (e.g., a subdistrict‐level health centre). Thus, communities encompass a wide range of stakeholders, including caregivers, health service providers, and influential community members such as religious or other traditional leaders. Therefore, our review will include any intervention that is directed towards any of the above types of community members. Interventions that target higher levels of the health system—such as a programme to improve state‐level officials' use of immunisation‐related data—will be excluded.

#### 1.2.2 What is community participation?

We define community participation as a process wherein communities are included, to various degrees, in planning, decision‐making and implementation of activities that directly impact them (Stuart, 2017). The Spectrum of Public Participation, developed by the International Association for Public Participation (iap2.org), identifies five levels of public (community) participation, ranging from *informing* communities to *empowering* them (Table 1).

**Table 1:** Spectrum of public participation

Level of involvement	Description
Inform	Reaching out to/informing the community about or as a part of an intervention
Consult	Consulting the community as part of a process to develop an intervention/programme, or build community awareness and understanding around an intervention
Involve	Involving the community through a range of mechanisms to ensure that issues and concerns are understood and considered as part of the decision‐making process
Collaborate	Collaborating with the community by developing partnerships to formulate and deliver an intervention
Empower	Shared leadership/empowering the community to make decisions and to implement and manage change.

The levels defined above refer to degrees of participation rather than types of intervention activities—indeed, these participation levels cut across intervention activities. For example, an intervention to inform or educate community members about the importance of immunisation could engage communities at the *inform* level (if communities are merely told that an informational campaign will be introduced in their area), at the *empower* level (if community members direct and manage all aspects of designing and implementing the informational campaign), or at any level in between. Similarly, interventions to motivate community members or increase health provider capacity could engage communities at any of these levels. In addition, a programme might also engage different community members at different levels (for different components of the same programme) or the level of engagement for the same set of participants may change over the course of a programme (Hardy, 2015).

However, it is important to note that the participation spectrum does not assume one level of participation to be better than the others or imply that the deeper the level of engagement, the better. In other words, the spectrum does not classify the participation levels into a hierarchy. Relatedly, the *quality* of engagement is distinct from the *level* of involvement—that is, although the spectrum is arranged from less to more substantive, the fact that interactions are more substantive does not always mean they are of higher quality. Thus, an intervention at one of the higher levels of involvement may still be deficient in the way it incorporates community input and feedback because of poor design or implementation. For example, an intervention could make efforts to understand community concerns and consider them in decision‐making, and thus be classified as an *involve*‐level programme. But those efforts to understand may be weak, or community concerns may be considered but ultimately given minimal weight in decision‐making.

It is also important to note that the spectrum aims to capture a wide range of ways that intervention implementers can interact with communities. Although simply informing a community that an intervention is being implemented is arguably not participatory (and hence, interventions at the inform level are arguably not “community participation” interventions), we rely on the Spectrum of Public Participation because it is an established framework for understanding community engagement. In particular, including interventions at the *inform* level in our review will allow us to assess the hypothesis, implicit in the design of many community‐engagement interventions, that informing communities is an insufficient level of engagement to effect change.

### 1.3 How the interventions might work

A 2015 3ie scoping paper (Sabarwal et al., 2015) systematically mapped the literature on immunisation interventions and also consulted several programme managers and policy experts to understand why community participation could be the key to improving immunisation outcomes for children in areas where the coverage has stagnated or declined or that are hard to reach. The findings from the scoping study indicate that working with or engaging communities could help develop an understanding of the context, target population, problems and barriers, and lead to identification of contextually relevant solutions and desired outcomes, and mobilising community support for them. In addition, existing literature on behaviour change (Bicchieri & Xiao, 2009; Reynolds, Subašić, & Tindall, 2015) highlights the importance of recognising that individuals usually function under the influence of social norms, that is, they respond to their peers and community, and while activities such as information and education campaigns might have some influence, individuals might feel bound by collective decisions (Riedy, 2012). The role of peers and of social norms in shaping attitudes towards vaccination is particularly important given that vaccine hesitancy has been documented in countries of all income levels (although it takes very different forms in different countries; see Dubé, Gagnon, Nickels, Jeram, & Schuster, 2014). Moreover, there is systematic evidence from both high‐income countries (HICs; O'Mara‐Eves et al., 2013) and LMICs (De Buck et al., 2017) that community engagement can be an effective model of modifying health behaviours in particular. Hence, community engagement could be a very important determinant of success or failure of an intervention aimed at improving immunisation coverage.

It is unlikely that any single theory of change would be able to capture all the different ways in which community participation (across the spectrum) affects immunisation outcomes. In particular, there is no strict correspondence between levels or types of engagement and specific intervention activities. For example, consider a proposed programme that aims to provide reminders to caregivers for immunisation. At less intensive levels of community participation, decisions related to the design and execution of the programme may have been undertaken by the sponsoring agency with limited inputs from the community. On the other hand, a similar programme can also result from conversations with the community, where members identify access to immunisation as a barrier because caregivers do not know when and where to take their children for immunisation, while at the same time the delivery of services is irregular. Additionally, the same participation process may also be leveraged to seek more substantive inputs from community members, including the frontline health workers, for designing the mode and content of reminders, targeting the hardest‐to‐reach households, and for mobilising the community at large. This example highlights how different intensity levels of community participation could give rise to the same activity involving reminders, which in turn could lead to improvement in access to immunisation services and an eventual increase in immunisation rates.

Importantly, the theory of change linking community participation to immunisation outcomes does not specify a precise causal mechanism whereby specific intervention activities yield specific behavioural responses and outcomes. Rather, the theory assumes that different barriers to immunisation exist in different communities, and that different categories of activities will be best suited to addressing those barriers. The underlying logic of using community participation to increase immunisation coverage is that engaging communities can be an effective way to (a) identify activities that will address the immunisation barriers extant in that community, and (b) implement those activities in ways that are appropriate for the local context.

**Figure 1:**
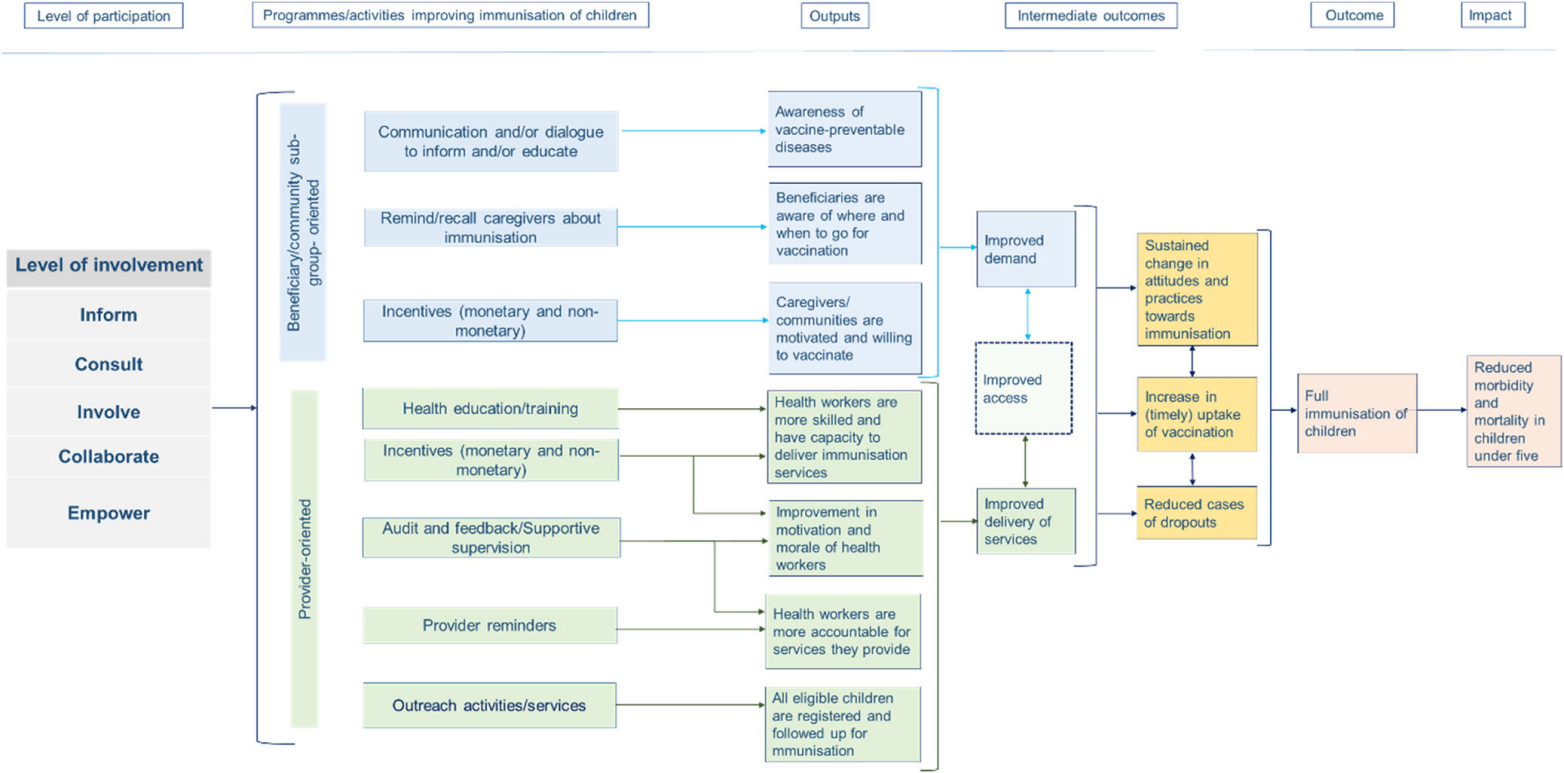
Simplified intervention theory of change

Figure 1 provides a simplified theory of change for how various types of immunisation activities, grounded in different levels of community participation, might lead to improved immunisation outcomes for children. The activities are broadly classified as two types: beneficiary/community‐ and provider‐oriented. These activities lead to outputs and outcomes related to improvement in demand for, delivery of, and access to immunisation services. This ultimately leads to improved timely uptake of vaccination(s) and an increase in full immunisation rates. As noted above, the precise causal mechanisms that begin with community engagement and end with improved immunisation rates will differ depending on the level of participation utilised and the nature of the activities selected; consequently, the theory of change does not depict the full details of each involvement level's mode of action. However, note that the theory does rely on the assumption that in order to produce the desired results, specific expertise, commitment, and resources will be required to engage the community effectively at the intended level of participation. The details of the theory of change—and in particular, how different levels of involvement will trigger different causal sequences—will be further developed in the course of the review and evidence synthesis.

#### 1.3.1 Why it is important to do the review

There are a number of existing SRs that address the effectiveness of interventions to increase immunisation coverage. However, none of these reviews are adequate for the purpose of guiding policymakers in deciding whether and how to pursue strategies that substantively involve various members of the community to address barriers all along the causal chain leading to vaccination delivery. We also believe it is important to have a review specific to LMICs. Many LMICs face similar barriers to achieving universal immunisation coverage, such as low rates of institutional births and poor infrastructure, which create logistical challenges for maintaining vaccination stocks and cold chains. In addition, health professionals in LMICs typically have lower literacy and skill levels which presents challenges for the delivery of quality immunisation services (Abejirinde, Ilozumba, Marchal, Zweekhorst, & Dieleman, 2018; Rowe et al., 2018). As these specific barriers are largely absent in HICs, reviews in which much of the evidence comes from HICs are of limited relevance to the needs of policymakers in LMICs.

Below we list 10 existing reviews and briefly document why they do not already accomplish the goals of our proposed review; see “References” section for full references.

Batt et al. (2004): This review covers only grey literature and is nearly 15 years old.

- Glenton et al. (2011): This review covers the use of lay health workers for increasing immunisation rates. While this overlaps with our proposed focus, our review will encompass other types of interventions that encourage community participation beyond those involving lay health workers (e.g., interventions aimed at community leaders and caregivers). In addition, only about half of the studies included in this review were conducted in LMICs.
- Kaufman et al. (2018): While the interventions covered in this review overlap with those in our proposed SR, there are key differences in scope. Kaufman et al.'s review covers only face‐to‐face interventions, whereas our review will include studies using any form of communicating with community members, including mass media and ICT (information and communication technology) modalities. In addition, 7 of 10 studies included in this review were conducted in HICs.
- Lassi and Bhutta (2015): Similar to our proposed review, the scope of Lassi et al.'s review is related to both community engagement as an intervention strategy and child health as an outcome. However, Lassi et al.'s review focuses on neonatal health and thus excludes many immunisation‐related interventions.
- Mureed et al. (2015): The reporting in this review is limited so it is not clear what interventions were included in the scope of the review. In addition, the review did not include grey or non‐English literature and lacks key policy‐relevant components such as causal path analysis and cost‐effectiveness information.
- Oyo‐Ita et al. (2016): This review is comprehensive and recent, covering a broad range of interventions targeting DPT immunisation in LMICs. However, this review covers only interventions that target either caregivers or health service providers, whereas our review will also include interventions aimed primarily at other community members (such as religious or other community leaders). Thus, an intervention that aimed to improve immunisation coverage by enlisting influential community members (who may not have young children themselves) to spread information about the importance of immunisation would not meet the intervention criteria reported in Oyo‐Ita et al.'s review, but would be included in ours. In addition, the literature in this area is expanding rapidly, so we anticipate that there will be a substantial amount of new evidence to be included in our review.^1^ Moreover, we plan to look at outcomes beyond DPT3 in our review and use an analysis framework focusing on levels of community participation which is different from the one used in this review.
- Pegurri et al. (2005): This review did not include grey literature and is over 10 years old.
- Ryman et al. (2008): This review is comprehensive and targets many of the same interventions as our proposed review (though without the specific focus on community‐participation strategies). However, it is now 10 years old.
- Saeterdal et al. (2014): This review is relatively recent (and we understand an update will likely be published soon) and focuses specifically on interventions targeting communities. However, the interventions covered in this review address only one of the barriers to vaccination delivery, viz., lack of knowledge or information. In contrast, our review will include interventions aimed at barriers all along the causal chain leading to vaccine delivery. For example, interventions that aim to *motivate* (caregivers to vaccinate or health workers to strengthen outreach efforts) would not fall under Saeterdal et al.'s scope but would be included in ours. In addition, our review will include multifaceted interventions, whereas the Saeterdal et al. review will include such interventions only if the effects of the information/education component can be isolated.^2^ Thus, while Saeterdal et al.'s review did not include studies such as Bolam et al. (1998) or Ryman et al. (2011), these studies would likely meet the intervention criteria for our review.^3^
- Shea et al. (2009): This review is nearly 10 years old and focuses exclusively on demand‐side interventions, whereas our proposed review will cover both demand‐ and supply‐side interventions.

Even though immunisation coverage in LMICs has increased in recent years, many WHO‐member countries still struggle to reach the target of 90% coverage for DPT and provide equitable access to life‐saving vaccines (WHO, 2017a). Even with strengthened routine immunisation programmes, marginalised and vulnerable communities—which may also be geographically or socially secluded—are susceptible to being left out. Increasingly, international and national policy frameworks emphasise community participation or engagement to increase immunisation coverage and reach the hardest‐to‐reach (UNICEF, n.d.; WHO, 2015, 2017a, 2017b). This includes educating community members about immunisation and its importance, getting their feedback on developing viable solutions to improving immunisation services, motivating them to take action to improve coverage, collaborating with them to identify and resolve issues that contribute to low immunisation rates, and empowering them to develop and improve systems for immunisation service delivery that fit the local context.

For instance, in 2011 the Ethiopian government launched a social mobilisation scheme, the Health Development Army, organising women from rural communities into volunteer networks to promote proper maternal and child health practices, including immunisation (Adamsu & Salama, 2014). Similarly, in 2014, the central government of India launched Mission Indradhanush (MI), which besides involving health administration at various levels, also involves community‐level workers to enlist beneficiaries in areas where routine immunisation coverage is weak. MI is also mobilising communities using communication strategies to improve immunisation (Ministry of Health and Family Welfare, 2016). Several other countries and international organisations have also adopted similar policies to strengthen planning, delivery and uptake of health services in order to reach the children, especially in the most vulnerable and marginalised populations. Reflecting the demand for evidence on the effectiveness of these approaches, the Gates Foundation, in collaboration with the International Initiative for Impact Evaluation (3ie), has commissioned a series of impact evaluations on immunisation interventions with a focus on community participation.^4^

## 2 OBJECTIVES

The objective of this review is to collect and synthesise all existing relevant evidence on the use of community participation strategies to improve immunisation outcomes in LMICs. In particular, we aim to answer the following five questions:

1. What evidence exists regarding the effectiveness of community participation interventions in improving routine immunisation coverage of children in LMICs?
2. Is there evidence for heterogeneous effects of community participation strategies (i.e., does effectiveness vary by region, population, gender or programme implementation)?
3. What intervention and implementation features are associated with relative success and failure in improving childhood routine immunisation outcomes?
4. What are the contextual barriers to, and facilitators of, the effectiveness of community participation in routine immunisation interventions?
5. What is the cost‐effectiveness of different community participation interventions in improving children routine immunisation outcomes?

In addition, we aim to assess whether there is any evidence of adverse effects of community participation interventions.

## 3 METHODOLOGY

Our review will follow Cochrane and Campbell Collaboration guidelines on conducting SRs. We will conduct a broad, systematic search, apply consistent criteria to the screening of studies for inclusion, and, where appropriate, synthesise studies through meta‐analysis. We will present the findings of the review in forest plots and a “Summary of Findings” table.

Items retrieved through the search will be imported to EPPI‐Reviewer 4 and independently screened by two trained reviewers at the title/abstract and full‐text levels. At the title/abstract level, we will use EPPI‐Reviewer's machine learning and text mining features to prioritise citations that are likely to be included and to exclude items that are very unlikely to be included (O'Mara‐Eves, Thomas, McNaught, Miwa, & Ananiadou, 2015; Thomas, McNaught, & Ananiadou, 2011). We will begin by manually screening 1,000 random records to train the machine learning algorithm, and have EPPI‐Reviewer order the remaining records according to their probability of inclusion as determined by EPPI‐Reviewer's algorithm. We will then set the EPPI‐Reviewer algorithm to learn continuously as we screen more records, and to continually re‐order remaining records according to their probability of inclusion. Thus, as we screen from the top of the list, records with low probabilities of inclusion will continue to be pushed further to the bottom of the list. When we reach a point where we exclude 500 consecutive records, we will automatically exclude the remaining titles (i.e., those with an even lower probability of inclusion than the 500 consecutive exclusions). We will, however, screen a random sample of the automatically excluded records to verify the accuracy of the algorithm.

Full‐text screening will be conducted by two independent reviewers, with disagreements resolved through discussion (and the lead author making final decisions on any contested cases). We will document the screening process so that a PRISMA flow chart and a table of characteristics of excluded studies can be provided in the review.

### 3.1 Criteria for including and excluding studies

#### 3.1.1 Types of study designs

The review will include experimental and quasiexperimental studies that estimate the causal impact of an intervention, as compared to usual practice, by establishing a counterfactual. The review will include studies with the following designs:

- Randomised controlled trials
- Regression discontinuity designs
- Instrumental variables estimation
- Statistical matching (e.g., propensity score matching)
- Difference‐in‐differences
- Fixed effects estimation
- Interrupted time series analysis

We include both experimental and quasiexperimental designs because randomisation is often impractical or unethical in the context of development interventions. The above designs are all established methods for drawing causal inferences about an intervention's effects (Shadish, Cook, & Campbell, 2002). There will be no inclusion restrictions based on study publication status.

We will not impose any restrictions based on language of publication. Between members of the review team and consultants, we will have the capacity to screen and extract data from studies in Chinese, Spanish, Portuguese and French. If our search retrieves potentially relevant studies in other languages, we will attempt to find qualified reviewers to screen and, if they meet our inclusion criteria, extract data from these studies.

The review will include only studies that measure the *effectiveness* of an intervention, rather than studies of *efficacy*. Because studies fall along a continuum from efficacy to effectiveness, rather than fitting cleanly into one category or the other (Singal, Higgins, & Waljee, 2014; Thorpe et al., 2009), we will not have precise determinative criteria to distinguish efficacy from effectiveness studies. Rather, we will use a set of questions to guide the decision of whether to classify a study as an efficacy trial (and hence ineligible for inclusion). The more a study exhibits the characteristics described in the questions below, the stronger will be the case for classifying it as an efficacy trial.^5^ Reviewers will be trained to flag any unclear cases for further review and discussion, with the lead author making final decisions on any contested cases.

#### 3.1.2 Research objective

(1) Does the study aim to determine how an intervention (e.g., technology, treatment, procedure or service) functions under ideal conditions, as opposed to how the intervention functions under “real‐world” conditions (i.e., approximating the conditions that would inhere in a large‐scale rollout of the intervention)?

#### 3.1.3 Population

(2) Are the participants likely unrepresentative of the general population? For example, are strict inclusion and exclusion criteria used to enrol a relatively homogenous population, which may limit the generalisability of the results?

#### 3.1.4 Providers

(3) Is the intervention primarily delivered by researchers, rather than people who might be expected to deliver the intervention under large‐scale rollout conditions (e.g., health workers, community leaders, or NGOs)?

Because the distinction between efficacy and effectiveness is a subtle one, we do not anticipate that reviewers will be able to determine which category a study falls into based on the title or abstract. Thus, only the full‐text screening protocol will include questions related to this distinction.

#### 3.1.5 Types of participants

For this review, we will include studies evaluating programmes targeted at rural, peri‐urban and urban populations to increase immunisation of children under five in LMICs (where a country's income status is determined by its World Bank classification at the time an intervention was carried out). All interventions that do not focus on immunisation outcomes for children (e.g., vaccination campaigns targeting adults) will be excluded. We will also exclude studies from high income countries, as the evidence from these countries will have limited applicability to the contextual factors prevalent in LMICs. As for the participants, while our review will focus on interventions targeting community members, interventions need not target the community as a whole; it is sufficient that an intervention targets any subgroup of a community as defined above. When only a subset of the participants/beneficiaries in a study are eligible (e.g., a study evaluating an intervention's effects on both childhood and adult vaccination), we will include the study only if the study reports outcomes separately for the eligible participants/beneficiaries.

#### 3.1.6 Types of interventions

Any intervention involving some degree of community participation, where our definitions of “community” and “participation” are given above, will be eligible for inclusion. The types of relevant interventions include, but are not limited to:

Interventions to promote communication and community dialogue about routine immunisation

- Interventions to promote community participation in the design of activities to encourage routine immunisation
- Interventions to promote community participation in designing and implementing routine immunisation delivery services
- Interventions to improve monitoring and accountability mechanisms for routine immunisation‐related activities
- Peer recognition and monetary incentives to improve routine immunisation outcomes for children

Interventions that do not engage communities as defined above (i.e., people served by a given primary health facility) will be excluded. Also excluded will be interventions that do not involve any of the participation levels in the Spectrum of Community Participation (e.g., those that do not even inform communities about an intervention).^6^

#### 3.1.7 Types of outcome measures

To be included, studies must assess at least one of the primary or secondary outcomes identified below.

##### Primary outcomes

- Full immunisation coverage (FIC)
- Antigen specific immunisation coverage (e.g., DPT3 or Penta 3 coverage, measles coverage)
- Timely uptake of vaccines

##### Secondary outcomes

- DPT 1/Penta 1—DPT 3/Penta 3 dropout rates
- OPV1—OPV3 dropout rates
- Mortality
- Morbidity
- Knowledge, attitudes and practices related to immunisation
  – Increase in awareness
  – Increase in health seeking activities

- Access to immunisation (distance to the primary health centre)
- Cost‐effectiveness of interventions
  – Cost per additional child fully immunised
  – Cost per additional child not dropping out from penta 1 to penta 3
  – Cost per additional DALY averted
  – Cost per additional death averted

- Partial routine immunisation for children
- No routine immunisation for children
- Reporting heterogeneous impacts (by gender, socioeconomic indicators, etc.)

Although outcomes related to coverage and timeliness of vaccinations are the primary focus of this review, a wide range of additional outcomes are included to capture necessary upstream conditions (e.g., demand for and access to immunisation services) and key downstream effects (e.g., morbidity and mortality) of the primary outcomes. In addition, although some outcome measures might differ in degree (full, partial or no routine immunisation) or might be a subset of each other (dropout after DPT1/Penta 1 will be categorised as partially immunised), the outcomes are wide ranging to account for the differences and preferences of authors in reporting them.

Both official health records (including vaccination cards) and parent recall will be considered acceptable measures of immunisation coverage. If a study reports both types of measures, we will extract data on both. We will note which measure the authors believe to be more reliable and treat this as the default “true” measure of the outcome. However, we will conduct sensitivity analysis to determine whether using a different coverage measure significantly affects the results of our analysis.

Although there are standardised tools for measuring constructs like parental attitudes towards vaccination (e.g., Oladejo et al., 2016; Shapiro et al., 2018), these have not yet been universally adopted or validated for populations of interest to this review (e.g., rural populations in LMICs). Therefore, we will defer to authors' definitions of these constructs and their instruments for measuring them.

#### 3.1.8 Duration of follow‐up

Because the timeline for administering most childhood vaccinations is short (most should be administered in the first 14 weeks after birth), there will be no restrictions on duration of follow‐up.

#### 3.1.9 Types of settings

As noted above, studies must have been conducted in a LMIC to qualify for inclusion. Apart from this, there will be no restrictions on types of settings. We anticipate that most studies will cover rural areas, but peri‐urban and urban settings are also eligible for inclusion.

Taking into account all of the above criteria, our scoping work has identified the following studies as ones that would likely meet our criteria and be included in the review:

- Banerjee et al. (2010)
- Bolam et al. (1998)
- Gibson et al. (2017)
- Habib et al. (2017)
- Robertson et al. (2013)
- Ryman et al. (2011)
- Uddin et al. (2016)

### 3.2 Search strategy

We will conduct a comprehensive search to identify as much relevant literature as possible. Appendix 1 includes a draft set of search terms to be used in electronic searches, and an example full search strategy for a key database (MEDLINE). The precise search strings and logic (e.g., index terms, truncation operators) will be adapted for each database. The review will document the full search strategy, including the specific strings used for each database. We will ensure that information on errata is included in results downloaded from electronic databases (hosts), so that errata can be consulted for included studies.

We will conduct electronic searches of the following databases of published sources:

- MEDLINE
- CAB Global Health
- EMBASE
- Cochrane Controlled Trials Register (CENTRAL)
- CINAHL
- PsycINFO
- Popline
- Africa‐wide information
- Academic search complete
- Scopus
- Campbell Library

To identify relevant grey literature, we will search the following databases and websites (some of which contain a mixture of published and grey literature):

- Google Scholar
- EconLit
- IDEAS/RePEc
- WHO Global Index Medicus
- Pascal‐Francis
- Open‐Grey
- Grey Literature Report
- Social Science Research Network (SSRN)
- Eldis
- GAVI
- Epistemonikos
- Innovations for Poverty Action (IPA)
- Abdul Latif Jameel Poverty Action Lab (J‐PAL)
- 3ie Impact Evaluation Repository
- 3ie Systematic Review Repository
- Registry of International Development Impact Evaluations (RIDIE)
- Global Development Network
- World Bank Development Impact Evaluation (DIME) and Impact Evaluation Policy Papers
- Inter‐American Development Bank
- Center for Global Development
- Center for Effective Global Action (CEGA)
- DFID Research for Development (R4D)
- USAID

In addition, we will be working with a team of researchers from Hong Kong University, who are experienced in information retrieval, to translate the search strategy and conduct searches of the following databases:

- Web of Science TM—Chinese Science Citation Database SM
- The China National Knowledge Infrastructure (CNKI)
- WanFang Med Online

We will also undertake hand searching of select journals to identify relevant papers published in the last 12 months that may not yet have been indexed. The full list of relevant journals will be developed using Journal Citation Reports and with input from sector experts and the information specialist (J. E.). The list will be finalised after the database search results have been screened. Journals to be hand searched will likely include: *The Lancet, BMJ, Vaccine, Bulletin of the World Health Organization, Journal of Tropical Medicine and Hygiene, Tropical Medicine and International Health, Journal of Development Economics, Journal of Development Effectiveness*, and *World Development*.

Once we have identified studies to be included, we will review references in included studies for additional sources, as well as search (using Google Scholar) for sources that cite included studies. Although the review will include only primary studies, we will also conduct a separate search for other relevant SRs. We will have a screening protocol for these reviews to identify those relevant to our research questions, and we will check the references of these reviews for additional sources.^7^ We will also share our included studies list with experts in the field and request them to identify any omissions.

In addition to an assessment of the effectiveness of immunisation interventions, our review will also include cost‐effectiveness analysis (Dhaliwal, Duflo, Glennerster, & Tulloch, 2011), as well as causal chain analysis (Kneale, Thomas, Bangpan, Waddington, & Gough, 2018) to identify facilitators and inhibitors of intervention effectiveness. To identify sources relevant to these analyses, we will conduct a targeted search for additional information on the interventions included in the effectiveness review, including cost information, process evaluations, and feasibility studies. We will use citation tracking to identify companion papers to included impact evaluations, as well as database and website searches using names of programmes and authors of included studies. We will also contact authors and implementing agencies to request additional project information if required.

We will conduct the first round of search shortly after the protocol is approved. Shortly before we begin to write up our findings in a SR report, we will re‐run the main electronic searches to identify any relevant papers that have been published or indexed while the SR was in progress. We will screen the newly‐retrieved studies and those that meet the eligibility criteria will be included in the review.

### 3.3 Description of methods used in primary research

We anticipate that most studies will use either a randomised controlled trial or difference‐in‐differences methods, but we will include studies employing any of the designs enumerated above in “Types of study designs”.

### 3.4 Criteria for determination of independent findings

Where more than one paper or report is identified on a single study, we will choose one as the “main” paper. A study may be designated as the main paper because it reports on our primary (as opposed to secondary) outcomes of interest or because it contains more detailed reporting of results. When multiple papers report different results on an identical outcome, we will contact the authors to enquire about the differences and choose the results that more accurately reflect the impact of the intervention as relevant to our research questions. If contacting the author does not yield a clear decision, we will use results from the paper with the latest publication date. Other papers from a study (besides the main paper) will be considered “secondary reports.” We will use these secondary reports to provide additional information about that one study, which may include outcome measures not reported in the main paper or supplementary information about implementation or costs.

Where information is collected on the same intervention for different outcomes at the same or different periods of time, we will extract information on the full range of outcomes over time. We will identify the most common follow‐up period and include the follow up measures that match this most closely in the meta‐analysis. Where multiple outcomes are reported from different specifications, we will select the specification with the lowest risk of bias in attributing impact, for example, the most appropriately specified outcomes equation. When studies include multiple outcome measures to assess related outcome constructs (e.g., measuring access to immunisation services through both number of days when health workers are present at health facilities and number of days when vaccines are stocked), we will select the outcome that appears to most accurately reflect the outcome construct of interest.

### 3.5 Critical appraisal

#### 3.5.1 Assessment of risk of bias in experimental and quasiexperimental studies (Review Questions 1–2)

We will assess risk of bias based on categories of bias recommended by the Cochrane Non‐Randomised Studies Group and procedures recommended by Waddington et al. (2017). These tools have been developed to assess the risk of bias for a range of quasiexperimental studies, as well as experimental studies. We will assess risk of bias based on the following criteria, coding each paper as “Yes,” “Probably Yes,” “Probably No,” “No” and “Unclear” according to how they address each domain:

1. Randomisation
2. Quasiexperimental design with properly constructed counterfactual
3. Spillovers/contamination
4. Outcome reporting
5. Analysis reporting
6. Performance
7. Attrition

The risk of bias for each included study will be conducted by two independent reviewers, with disagreements resolved through discussion (and the lead author making final decisions on any contested cases). We will report the results of the assessment for each of the assessed criteria for each study, and note the source of each judgement (e.g., page numbers of included studies where information is reported).

In addition, we will attempt to explore if there are systematic differences in outcomes between primary studies with different risk of bias. If meta‐analysis is feasible, we will conduct sensitivity analysis to assess the robustness of the results to the risk of bias in included studies.

#### 3.5.2 Critical appraisal of qualitative studies, process evaluations and project documents (Review Questions 3 and 4)

To address Review Questions 3 and 4, we will be drawing on a broader range of sources beyond impact evaluations, including qualitative studies, process evaluations and project documents. We will adopt different approaches to appraise the different types of documents, as outlined below.

We will assess the quality of included qualitative studies and descriptive quantitative studies using an adapted version of the Critical Appraisal Skills Programme checklist (CASP, 2018) developed by Snilstveit et al. (2015), making judgements on the adequacy of reporting, data collection, presentation, analysis and conclusions drawn. The draft checklist is included in Appendix 2, which may be adapted following further piloting.

For critical appraisal of process evaluations, we will draw on and adapt several resources. Rigorous process evaluations require reliable data from a representative sample, so assessment of sampling and methods of data collection are obvious issues to consider (Snilstveit et al. 2015), along with discussions of the assumptions underlying the evaluation and the transferability of results (Jimenez et al., 2018). We will draw on the above sources, along with existing guidelines for process evaluations (Scriven, 2015) and the CASP checklist to create a quality appraisal checklist suited to process evaluations.

Project documents provide information about planned, ongoing or completed interventions and we will be using them in our review to get sufficient information about the context and design of the interventions included in our review. As this information is descriptive we will not formally appraise the quality of such documents.

#### 3.5.3 Critical appraisal of cost evidence (Review Question 5)

For appraisal of cost evidence we will mainly draw on the approach taken in Doocy and Tappis (2017). They adapt two guides to the use and appraisal of cost evidence; the German Federal Ministry for Economic Cooperation and Development's Tools and Methods for Evaluating the Efficiency of Development Interventions (Palenberg, 2011) and the Campbell Collaboration Economic Methods Policy Brief (Shemilt et al., 2008).

### 3.6 Details of study coding categories

Studies that meet the inclusion criteria will go through a coding procedure to: record information about the study's publication, setting, and design; extract data on reported results; and assess risk of bias. All coding will be done by two independent, trained coders, with discrepancies resolved through discussion (and the lead author making final decisions on any contested cases). Table 2 presents a draft of the coding fields and instructions.

**Table 2:** Draft coding tool

Coding field	Description/coding instructions
Study title	Use only the English version of the publication's main title. If paper is not written in English and has the title translated, use the translated version of the title. If the publication does not provide an English version, include the title in its original language
Foreign title	When publication is not written in English, code the original title using original accents and special characters
Language	Select full text language that applies: English, French, Spanish, Portuguese, or Chinese
Authors	Enter all study authors
Publication type	Whether the study is a journal article, working paper, report, and so forth
Publication source	The journal, working paper series, or institution publishing the study
Year	Input the year when the print version of the study was published. If the publication is online‐only, then input the date the publication appeared online
Country or countries	The country or countries where the study was conducted
Setting	Rural, urban, or peri‐urban
Equity focus	How does this study consider gender and/or equity?
Equity dimension	Which dimension(s) of gender and/or equity does the intervention target?
Equity description	Open answer—provide a description of how the study considers gender and equity, and for which population to corroborate answers above (include page numbers where relevant)
Adverse effects	Input any potential adverse effects of the intervention that the study reports on. If study does not report on any potential adverse effects, code as “Not applicable”
Evaluation design	The general evaluation design (i.e., experimental or quasiexperimental)
Evaluation method	The specific method used to evaluate impact (mainly applicable to quasiexperimental studies—regression discontinuity, instrumental variables, etc.)
Unit of observation	The unit(s) of observation/analysis used in the study, for example, individual, household, village, and so forth
Programme name	Input the name of the project/programme being evaluated (if any)
Implementing agency	Input the name of the agency or agencies implementing the programme
Programme funding agency	Input the name of the agency or agencies funding the programme (note: this is not the same as organisations that fund the research of the evaluation)
Research funding agency	Input the name of the agency or agencies funding the research (note: this is not the same as organisations that fund the programme)
Number of study arms	The number of separate study arms (e.g., control + two versions of treatment = 3 arms).
Control condition description	Briefly describe the “usual practice” that the control group receives while the treatment group is receiving the intervention
Intervention A: primary targets	Who are the *primary* targets of the intervention (i.e., those who have substantive interactions with programme implementers from outside the community)? For example, caregivers, health workers, or community leaders
Intervention A: secondary targets	Who are the *secondary* targets of the intervention (i.e., those who, as part of the intervention's design, have substantive interactions with the primary targets). For example, caregivers who receive information from community leaders, who are the primary targets as defined above. If there are no secondary targets, code as “Not applicable”
Intervention A: activities	Briefly describe the activities that comprise the intervention
Intervention A: level of involvement	Specify the intervention's level of involvement of the community (Inform, Consult, Involve, Collaborate or Empower). This should be the intervention's level of involvement as it was actually implemented (regardless of how it was designed)
Intervention A: quality of engagement	Specify whether the quality of engagement with the community was low, medium, high or cannot be determined from the reporting in the study
Intervention B: primary targets	Who are the *primary* targets of the intervention (i.e., those who have substantive interactions with programme implementers from outside the community)? For example, caregivers, health workers, or community leaders. If only one treatment arm, code as “Not applicable”
Intervention B: secondary targets	Who are the *secondary* targets of the intervention (i.e., those who, as part of the intervention's design, have substantive interactions with the primary targets). For example, caregivers who receive information from community leaders, who are the primary targets as defined above. If there are no secondary targets, or if there is only one treatment arm, code as “Not applicable”
Intervention B: activities	Briefly describe the activities that comprise the intervention. If only one treatment arm, code as “Not applicable”
Intervention B: level of involvement	Specify the intervention's level of involvement of the community (Inform, Consult, Involve, Collaborate or Empower). This should be the intervention's level of involvement as it was actually implemented (regardless of how it was designed). If only one treatment arm, code as “Not applicable”
Intervention B: quality of engagement	Specify whether the quality of engagement with the community was low, medium, high, or cannot be determined from the reporting in the study. If only one treatment arm, code as “Not applicable”
*Add rows for additional interventions as needed*	
Take‐up of intervention	Does the study report on the level of intervention take‐up?
Knowledge/awareness/attitudes	Does the study report on changes in participants' knowledge, awareness, or attitudes?
Access to immunisation services	Does the study report on changes in participants' access to immunisation services?
Health‐seeking behaviour	Does the study report on changes in participants' health‐seeking behaviour?
Timely uptake of vaccinations	Does the study report on changes in timely uptake of vaccinations?
DPT3/penta3 coverage	Does the study report on coverage of DPT3 or penta3 vaccines?
Measles coverage	Does the study report on coverage of measles vaccination?
Antigen‐specific coverage	Does the study report on immunisation coverage for other specific antigens?
DPT1—DPT3/penta1—penta3 dropouts	Does the study report on rate of dropouts from DPT1 or penta1 to DPT3 or penta3?
OPV1‐OPV3 dropouts	Does the study report on rate of dropouts from OPV1 to OPV3
Morbidity	Does the study report on morbidity outcomes?
Mortality	Does the study report on mortality outcomes?
Gender	Does the study report on differential impacts by gender?
Socioeconomic status	Does the study report on differential impacts by socioeconomic status (e.g., wealth quantile)?
Rural versus peri‐urban versus urban	Does the study report on differential impacts by rural/peri‐urban/urban population setting?
Other vulnerable group	Does the study report on differential impacts by any other vulnerable group?
Study *n*	Input the total sample size of the study
Control group *n*	Input the sample size of the control group
Treatment A *n*	Input the sample size of the first treatment arm
Treatment B *n*	Input the sample size of the second treatment arm. If there is only one treatment arm, code as “Not applicable”
Treatment C *n*	Input the sample size of the third treatment arm. If there are fewer than three treatment arms, code as “Not applicable”
Outcome 1 indicator	Input the first outcome indicator for which results are reported (e.g., DPT3 coverage, fully immunised, etc.)
Outcome 1: coefficient reported	Input the type of measure used to report the intervention's effects. Select from:
	Percentage change Risk ratio Odds ratio Difference in means
Outcome 1: confidence interval	Input the confidence interval using this format: [lower bound, upper bound]. If not reported, code as “Not specified”
Outcome 1: confidence level	Input the level of confidence for the confidence interval: (e.g., 90%, 95%, or 99%). If not reported, code as “Not specified”
Outcome 1: standard error	Input the reported standard error of the effect size. If not reported, code as “Not specified”
Outcome 1: significance level	Input the significance level of the effect size:
	0.10 if significant at 10% 0.05 if significant at 5% if significant at 1% Not significant
	If not reported, code as “not specified”
Outcome 1: Control group “yes”	The number of participants in the control group who attained the reported outcome (e.g., received DPT3)
Outcome 1: Control group “no”	The number of participants in the control group who did not attain the reported outcome (e.g., received DPT3)
Outcome 1: Treatment A “yes”	The number of participants in Treatment A who attained the reported outcome (e.g., received DPT3)
Outcome 1: Treatment A “no”	The number of participants in Treatment A who did not attain the reported outcome (e.g., received DPT3)
Outcome 1: Treatment B “yes”	The number of participants in Treatment B who attained the reported outcome (e.g., received DPT3). If there is only one treatment arm, code as “Not applicable”
Outcome 1: Treatment B “no”	The number of participants in Treatment B who did not attain the reported outcome (e.g., received DPT3). If there is only one treatment arm, code as “Not applicable”
Outcome 1: Treatment C “yes”	The number of participants in Treatment C who attained the reported outcome (e.g., received DPT3). If there are fewer than three treatment arms, code as “Not applicable”
Outcome 1: Treatment C “no”	The number of participants in Treatment C who did not attain the reported outcome (e.g., received DPT3). If there are fewer than three treatment arms, code as “Not applicable”
*Add rows for additional outcomes as needed*	
Cost per additional child fully immunised	Input the reported cost per additional child fully immunised. If not reported, code as “not applicable”
Cost per additional child not dropping out from DPT1 or penta1 to DPT3 or penta3	Input the reported cost per dropout prevented. If not reported, code as “not applicable”
Cost per DALY averted	Input the reported cost per DALY averted. If not reported, code as “not applicable”
Cost per additional death averted	Input the reported cost per additional death averted/life saved. If not reported, code as “not applicable”

### 3.7 Statistical procedures and conventions

We will use forest plots to display the distribution of effect sizes and confidence intervals for similar outcomes across included studies. Where studies are sufficiently similar in terms of their interventions, populations, and designs, we will use meta‐analysis to pool and analyse results. As recommended by the *Cochrane Handbook of Systematic Reviews* (section 9.4.4.1), we will weight studies in the meta‐analyses using the Mantel‐Haenszel method. Given that contextual factors are likely to vary significantly across studies, we plan to use random‐effects meta‐analysis. Where outcome measures are dichotomous (i.e., vaccination received or not), we will extract data and calculate log‐transformed risk ratios to derive standardised effect sizes across studies. For any continuous outcomes (e.g., knowledge about or attitudes towards immunisation), we will extract data and calculate the standardised mean difference (SMD), to which we will apply the Hedge's *g* transformation. We will conduct separate meta‐analyses for different immunisation‐related outcomes (e.g., DPT3 coverage and FIC). Meta‐analysis results will be presented in forest plots. We will check the forest plots against results from included studies to check that data have been extracted and coded appropriately (e.g., direction of effect has not accidentally been reversed).

When data cannot be extracted in a usable fashion from included papers, we will contact the authors to request the necessary data. If this fails, studies without usable data will be included in the review even if they cannot be included in the meta‐analyses.

Where cluster‐randomised trials have appropriately adjusted for cluster effects (e.g., by using clustered standard errors), we will rely on the effect sizes and standard errors reported in the studies. Where authors have not appropriately adjusted for clustering, we will make adjustments as recommended in the *Cochrane Handbook for Systematic Reviews* (Higgins, Eldridge, & Li, 2019) and related literature (e.g., Donner & Klar, 2002). When there are multiple treatment arms being compared to a single control arm (e.g., Treatment A vs. control and Treatment B vs. control), we will include only intervention and control groups that meet eligibility criteria. When multiple arms of the same study meet eligibility criteria, we will divide the control group sample size by the number of treatment arms to avoid double counting.

We will use the *Q, τ* ^2^, and *I* ^2^ statistics to analyse the heterogeneity of results across studies, and attempt to explain observed heterogeneity through moderator analysis, including subgroup analysis and, if applicable, meta‐regression. We will conduct moderator analyses to investigate sources of heterogeneity. We will use the framework of Lipsey (2009) which classifies moderators into three broad categories of extrinsic, methodological and substantive characteristics. We aim to include extrinsic variables such as funder and publication date; methodological variables such as study design and risk of bias; and substantive variables such as participant characteristics (gender, socioeconomic status), and context (country, geographical setting). While the exact set of moderators will be determined as we learn more about the characteristics of included studies, at a minimum we will (again, assuming meta‐analysis is feasible at all) present meta‐analyses that stratify by study design (experimental vs. quasiexperimental), by risk of bias, and by level of community involvement. In addition, we hope to be able to analyse the role of *quality* of engagement as a moderator, though we may be limited by the detail of reporting in included studies.

More generally, as we continue to refine our theory of change, we will identify key pathways in the causal chain and identify study characteristics that could plausibly make a difference to the functioning of these pathways.

If there are more than 10 studies reporting the same outcome that can be combined in a meta‐analysis, we will use funnel plots to examine risk of bias due to missing results (Page, Higgins, & Sterne, 2019).

To incorporate cost data and to undertake cost effectiveness analysis, we will largely follow the methodology in Dhaliwal et al. (2011). We expect that most of this data will not be found in the published papers, so we anticipate having to go back to authors to collect this data. Costs will include the costs of administration, targeting, training, monitoring, materials, users, and so forth. On impact size, we will focus on one outcome at a time (e.g., full immunisation), but also make sure to indicate if the programme affected other outcomes. Insignificant impacts will not be included in the cost‐effectiveness analysis.

We will use Review Manager 5 to complete and submit the review, while using Stata 14 for all statistical analyses, including the metan command for meta‐analyses.

### 3.8 Treatment of qualitative research

We will use qualitative research to supplement the findings of our quantitative synthesis. While we will not seek out all qualitative studies relating to community engagement and immunisation in LMICs, we will look for qualitative studies to provide additional information about the context and the implementation of interventions included in the quantitative synthesis. This may include feasibility studies, stakeholder analyses, formative evaluations, process evaluations, and project reports, among other documents. These sources will provide key inputs to our analysis of the facilitators and inhibitors of effective community engagement interventions.

## ROLES AND RESPONSIBILITIES

M. J., M. E., and A. B. have content knowledge in childhood immunisation programmes in low and middle income countries. M. J. and M. G. have statistical backgrounds and the statistical analysis will be led by M. J. M. G. has prior experience conducting systematic reviews and cost‐effectiveness analyses. M. E. and A. B. have prior experience with screening and coding data from studies. J. E. has expertise in information retrieval and will be leading the design and execution of the search.

## SOURCES OF SUPPORT

This work is supported by the International Initiative for Impact Evaluation (3ie).

## DECLARATIONS OF INTEREST

The International Initiative for Impact Evaluation (3ie) provides funding and technical assistance for seven ongoing impact evaluations of community engagement interventions for immunisation as a part of its immunisation evidence programme. This technical assistance includes, but is not limited to: reviewing study designs, analysis plans, and data collection instruments; advising research teams on how to improve study components and address challenges that arise during the course of the evaluation; and supporting grantees in engaging with stakeholders to promote uptake and use of evidence generated by the evaluations.

As members of 3ie staff, authors Monica Jain, Mark Engelbert, Marie Gaarder, and Avantika Bagai have all had varying levels of involvement in reviewing proposals for these evaluations and providing research teams with technical assistance. These SR authors therefore have a vested interest in the success and prominence of these studies, which fall under the scope of the proposed SR. This presents a conflict of interest in that the authors may apply different standards to these studies when reviewing them for inclusion in the SR or deciding how much weight to give them in the analysis.

However, there are several procedural safeguards and transparency measures in place that mitigate the risk this conflict of interest imposes. First, all candidate studies, including those funded by 3ie, will undergo a rigorous multi‐step screening process, including review at the title, abstract, and full‐text levels. To qualify for inclusion in the SR, a study must be judged to meet the inclusion criteria by two independent screeners who have reviewed the full text of the study. The screening protocol will be made publicly available, as will the protocol for analysing included studies.

Moreover, the screening for this SR will be conducted by independent consultants who, while paid by 3ie, will not be 3ie staff and will not have provided technical assistance or interacted with authors of 3ie‐funded studies. Finally, the authors will, upon request, provide full records of each round of screening, detailing the studies that were excluded and included at the title, abstract, and full‐text levels.

The authors have no financial interests in this area and have not published any prior reviews on the topic.

## PRELIMINARY TIMEFRAME

We plan to submit the review in or around February, 2020.

## PLANS FOR UPDATING THE REVIEW

Contingent on funding, this review will be updated two years after the original review is published. Monica Jain will lead the update. If for any reason we are unable to carry out this update, we will notify the International Development Coordinating Group and, if requested, help identify a suitable team of authors to update the review.

## Footnotes

^1^
A list of representative studies likely to be included in our review is provided in the “Methodology” section below, including several that have been published in the last two years.

^2^
Simon Lewin, personal communication.

^3^
Ryman et al. is listed among Saeterdal et al.'s excluded studies, with “Intervention” given as the reason for exclusion. Bolam et al. is not cited in Saeterdal et al.'s review.

^4^
See http://3ieimpact.org/en/funding/thematic-window/increasing-immunisation-thematic-window/

^5^
These criteria are adapted from Snilstveit et al. (2015, pp. 13–14), who in turn draw on Gartlehner et al. (2006) and Thorpe et al. (2009). See also Cameron and Mishra (2016).

^6^
Again, note that the *inform* level of community participation is distinct from the use of informational campaigns as part of an intervention. Thus, a vaccine information campaign that was deployed in a community (e.g., in the form of radio announcements), but about which no community members were informed ahead of time, would be excluded.

^7^
Note that, simultaneously with this SR, we will be completing an evidence gap map (EGM) on the same topic that will include systematic reviews. Thus, the reviews whose references we check will be those that are included in our EGM.

## APPENDIX 1 SEARCH STRATEGY

### Search terms

#### LMICs

1. (Afghanistan or Albania or Algeria or Angola or Argentina or Armenia or Armenian or Azerbaijan or Bangladesh or Benin or Byelarus or Byelorussian or Belarus or Belorussian or Belorussia or Belize or Bhutan or Bolivia or Bosnia or Herzegovina or Hercegovina or Botswana or Brazil or Bulgaria or Burkina Faso or Burkina Fasso or Upper Volta or Burundi or Urundi or Cambodia or Khmer Republic or Kampuchea or Cameroon or Cameroons or Cameron or Camerons or Cape Verde or Cabo Verde or Central African Republic or Chad or Tchad or China or Colombia or Comoros or Comoro Islands or Comores or Mayotte or Congo or Zaire or Costa Rica or Cote d'Ivoire or Ivory Coast or Cuba or Djibouti or French Somaliland or Dominica or Dominican Republic or East Timor or East Timur or Timor Leste or Ecuador or Egypt or United Arab Republic or El Salvador or Eritrea or Ethiopia or Fiji or Gabon or Gabonese Republic or Gambia or Gaza or Georgia Republic or Georgian Republic or Ghana or Grenada or Guatemala or Guinea or Guiana or Guyana or Haiti or Honduras or India or Maldives or Indonesia or Iran or Iraq or Jamaica or Jordan or Kazakhstan or Kazakh or Kenya or Kiribati or Korea or Kosovo or Kyrgyzstan or Kirghizia or Kyrgyz Republic or Kirghiz or Kirgizstan or Lao PDR or Laos or Lebanon or Lesotho or Basutoland or Liberia or Libya or Macedonia or Madagascar or Malagasy Republic or Malaysia or Malaya or Malay or Sabah or Sarawak or Malawi or Mali or Marshall Islands or Mauritania or Mauritius or Agalega Islands or Mexico or Micronesia or Middle East or Moldova or Moldovia or Moldovian or Mongolia or Montenegro or Morocco or Ifni or Mozambique or Myanmar or Myanma or Burma or Namibia or Nepal or Netherlands Antilles or Nicaragua or Niger or Nigeria or Muscat or Pakistan or Palau or Palestine or Panama or Paraguay or Peru or Philippines or Philipines or Phillipines or Phillippines or Papua New Guinea or Romania or Rumania or Roumania or Rwanda or Ruanda or Saint Lucia or St Lucia or Saint Vincent or St Vincent or Grenadines or Samoa or Samoan Islands or Navigator Island or Navigator Islands or Sao Tome or Senegal or Serbia or Montenegro or Seychelles or Sierra Leone or Sri Lanka or Solomon Islands or Somalia or Sudan or Suriname or Surinam or Swaziland or Eswatini or South Africa or Syria or Tajikistan or Tadzhikistan or Tadjikistan or Tadzhik or Tanzania or Thailand or Togo or Togolese Republic or Tonga or Tunisia or Turkey or Turkmenistan or Turkmen or Uganda or Ukraine or Uzbekistan or Uzbek or Vanuatu or New Hebrides or Venezuela or Vietnam or Viet Nam or West Bank or Yemen or Zambia or Zimbabwe or Rhodesia)
2. (Africa or Asia or Caribbean or West Indies or South America or Latin America or Central America)
3. ((developing or less* developed or least developed or under developed or underdeveloped or middle income or low* income or underserved or under served or deprived or poor* or resource limited or resource constrained) adj (countr* or nation? or population? or world or state* or emerging econom* or global south))
4. ((developing or less* developed or least developed or under developed or underdeveloped or middle income or low* income or resource limited or resource constrained) adj (economy or economies))
5. (low* adj (gdp or gnp or gross domestic or gross national))
6. (low adj3 middle adj3 countr*)
7. (lmic or lmics or third world or lami countr*)
8. transitional countr*
9. 1 OR 2 OR 3 OR 4 OR 5 OR 6 OR 7

#### Immunisation

1. (immuniz* or immunis* or vaccin* or inoculat* or innoculat* or immunotherap* or prophyla*)

#### Vaccination uptake and barriers to uptake

1. (uptake or up‐take or takeup or take‐up or decide* or decision* or barrier* or obstacle* or deter* or hesitan* or attitud* or knowledge or refus* or unwilling* or avoid* or adher* or persist* or continu* or distan* or travel* or remote or religio* or cultur* or taboo* or superstitio* or belie* or ((cultural* or social*) adj3 (norm* or practice*)) or confiden* or complacen* or access* or underserv* or poor or poverty or impoverished or "low‐income*" or "low‐resource*" or "low income*" or "low resource*" or "resource‐constrain*" or "resource* poor" or resource limited or dropout* or drop‐out* or default*)

#### Community engagement and participation

1. ((communit* or village* or local or rural or neighbo?rhood*) adj6 (health or develop* or participat* or engag* or partner or involv* or enlist* or enrol* or collaborat* or consult* or decid* or decision* or facilitat* or build* or manage* or own* or recruit or network* or stakeholder* or support or outreach or capacit* or mobili#ation or mobili#e or organi#ation or organi#e or inform* or educat* or instruct* or teach or empower* or train* or workshop* or leader* or service* or cent$3 or clinic*))

#### Young children and caregivers

1. (child* or infant* or newborn* or neonat* or prenatal or pre natal or antenatal or ante natal or baby or babies or toddler* or preschool* or parent* or mother* or father* or maternal or paternal or caregiver* or grandparent* or grandmother* or grandfather* or family member*)

#### Impact evaluation methods

1. (random* or experiment* or (match* adj2 (propensity or coarsened or covariate)) or "propensity score" or "difference in difference*" or "difference‐in‐difference*" or "differences in difference*" or "differences‐in‐difference*" or "double difference*" or "quasi‐experimental" or "quasi experimental" or "quasi‐experiment" or "quasi experiment" or ((estimator or counterfactual) and evaluation*) or "instrumental variable*" or (IV adj2 (estimation or approach)) or regression discontinuity or time series or segment* regression)

#### Cost‐effectiveness

1. (cost‐effective* or cost‐benefit)
2. ("life year" or "life years" or qaly* or daly*)
3. (economic* or cost*) adj6 (mortality or death* or markov)
4. ("cost minimi*" or "cost‐utilit*" or "economic evaluation*" or "economic review*" or "cost outcome" or "cost analys*" or "economic analys*" or "budget* impact analys*")
5. 1 OR 2 OR 3 OR 4

#### Example full search strategy

Below we present a draft of the full search strategy we will use to search MEDLINE. Note that in MEDLINE's syntax, terms with strokes (e.g., Immunisation/) denote Medical Subject Heading (MeSH) terms, while strings appended with “ti,ab,kw” will be searched in the title, abstract, and keyword fields of records in the database.

**Database: Ovid MEDLINE(R) and Epub Ahead of Print, In‐Process & Other Non‐Indexed Citations, Daily and Versions(R)**  < **1946 to February 07, 2019**>

Search Strategy:

1. (immuniz* or immunis* or vaccin* or inoculat* or innoculat* or immunotherap* or prophyla*).ti,ab,kw. (660952)
2. immunisation/or immunisation, passive/or immunisation schedule/or immunisation, secondary/or immunotherapy, active/or vaccination/or Immunisation Programs/or mass vaccination/(156772)
3. Tuberculosis Vaccines/or BCG Vaccine/or Diphtheria‐Tetanus Vaccine/or Meningococcal Vaccines/or Pertussis Vaccine/or Diphtheria‐Tetanus‐acellular Pertussis Vaccines/or Diphtheria‐Tetanus‐Pertussis Vaccine/or Diphtheria‐Tetanus Vaccine/or Measles Vaccine/or Mumps Vaccine/or Rubella Vaccine/or Measles‐Mumps‐Rubella Vaccine/or Poliovirus Vaccines/or Poliovirus Vaccine, Inactivated/or Poliovirus Vaccine, Oral/or Japanese Encephalitis Vaccines/or Rotavirus Vaccine/(49200)
4. or/1‐3 (708606)
5. developing countries.sh,kf. (82634)
6. (Africa or Asia or Caribbean or West Indies or South America or Latin America or Central America).ti,ab,kw. (196668)
7. Africa/or Asia/or Caribbean/or West Indies/or South America/or Latin America/or Central America/(72637)
8. (Africa or Central America or South America or Caribbean or Central Asia or Afghanistan or Albania or Algeria or Angola or Argentina or Armenia or Armenian or Azerbaijan or Bangladesh or Benin or Byelarus or Byelorussian or Belarus or Belorussian or Belorussia or Belize or Bhutan or Bolivia or Bosnia or Herzegovina or Hercegovina or Botswana or Brazil or Bulgaria or Burkina Faso or Burkina Fasso or Upper Volta or Burundi or Urundi or Cambodia or Khmer Republic or Kampuchea or Cameroon or Cameroons or Cameron or Camerons or Cape Verde or Cabo Verde or Central African Republic or Chad or Tchad or China or Colombia or Comoros or Comoro Islands or Comores or Mayotte or Congo or Zaire or Costa Rica or Cote d'Ivoire or Ivory Coast or Cuba or Djibouti or French Somaliland or Dominica or Dominican Republic or East Timor or East Timur or Timor Leste or Ecuador or Egypt or United Arab Republic or El Salvador or Eritrea or Ethiopia or Fiji or Gabon or Gabonese Republic or Gambia or Gaza or Georgia Republic or Georgian Republic or Ghana or Grenada or Guatemala or Guinea or Guiana or Guyana or Haiti or Honduras or India or Maldives or Indonesia or Iran or Iraq or Jamaica or Jordan or Kazakhstan or Kazakh or Kenya or Kiribati or Korea or Kosovo or Kyrgyzstan or Kirghizia or Kyrgyz Republic or Kirghiz or Kirgizstan or Lao PDR or Laos or Lebanon or Lesotho or Basutoland or Liberia or Libya or Macedonia or Madagascar or Malagasy Republic or Malaysia or Malaya or Malay or Sabah or Sarawak or Malawi or Mali or Marshall Islands or Mauritania or Mauritius or Agalega Islands or Mexico or Micronesia or Middle East or Moldova or Moldovia or Moldovian or Mongolia or Montenegro or Morocco or Ifni or Mozambique or Myanmar or Myanma or Burma or Namibia or Nepal or Netherlands Antilles or Nicaragua or Niger or Nigeria or Muscat or Pakistan or Palau or Palestine or Panama or Paraguay or Peru or Philippines or Philipines or Phillipines or Phillippines or Papua New Guinea or Romania or Rumania or Roumania or Rwanda or Ruanda or Saint Lucia or St Lucia or Saint Vincent or St Vincent or Grenadines or Samoa or Samoan Islands or Navigator Island or Navigator Islands or Sao Tome or Senegal or Serbia or Montenegro or Seychelles or Sierra Leone or Sri Lanka or Solomon Islands or Somalia or Sudan or Suriname or Surinam or Swaziland or Eswatini or South Africa or Syria or Tajikistan or Tadzhikistan or Tadjikistan or Tadzhik or Tanzania or Thailand or Togo or Togolese Republic or Tonga or Tunisia or Turkey or Turkmenistan or Turkmen or Uganda or Ukraine or Uzbekistan or Uzbek or Vanuatu or New Hebrides or Venezuela or Vietnam or Viet Nam or West Bank or Yemen or Zambia or Zimbabwe or Rhodesia).ti,ab,kw,sh. (1301719)
9. ((developing or less* developed or least developed or under developed or underdeveloped or middle income or low* income or underserved or under served or deprived or poor* or resource limited or resource constrained) adj (countr* or nation? or population? or world or state*)).ti,ab,kw. (89034)
10. ((developing or less* developed or least developed or under developed or underdeveloped or middle income or low* income or resource limited or resource constrained) adj (economy or economies)).ti,ab,kw. (463)
11. (low* adj (gdp or gnp or gross domestic or gross national)).ti,ab,kw. (226)
12. (low adj3 middle adj3 countr*).ti,ab,kw. (12151)
13. (lmic or lmics or third world or lami countr*).ti,ab,kw. (6251)
14. (transitional countr* or emerging econom* or global south).ti,ab,kw. (830)
15. or 6 or 7 or 8 or 9 or 10 or 11 or 12 or 13 or 14 (1458856)
16. and 15 (77905)
17. Health Campaigns/or Information Dissemination/or Information Centers/or Consumer Health Information/or Health Promotion/or Patient Education as Topic/or Social Marketing/or Marketing of health services/or Mass Media/or Health Knowledge, Attitudes, Practice/or Attitude/or Attitude to Health/or Health Literacy/or Information Literacy/or Access to Information/or Communication Barriers/or Information Seeking Behavior/or Decision Making/or Treatment Adherence and Compliance/or Patient Acceptance of Health Care/or Vaccination Refusal/or Patient Dropouts/or Patient Compliance/or Medication Adherence/or Poverty Areas/or Poverty/or Socioeconomic Factors/or Social Class/or Healthcare Disparities/or Health Equity/(723816)
18. (uptake or up‐take or takeup or take‐up or decide* or decision* or barrier* or obstacle* or deter* or hesitan* or attitud* or knowledge or refus* or unwilling* or avoid* or adher* or persist* or continu* or distan* or travel* or remote or religio* or cultur* or taboo* or superstitio* or belie* or ((cultural* or social*) adj3 (norm* or practice*)) or confiden* or complacen* or access* or underserv* or poor or poverty or impoverished or "low‐income*" or "low‐resource*" or "low income*" or "low resource*" or "resource‐constrain*" or "resource* poor" or resource limited or dropout* or drop‐out* or default*).ti,ab,kw. (8142575)
19. 17 or 18 (8471336)
20. ((communit* or village* or local or rural or neighbo?rhood* or urban or peri‐urban) adj6 (health or develop* or participat* or engag* or partner or involv* or enlist* or enrol* or collaborat* or consult* or decid* or decision* or facilitat* or build* or manage* or own* or recruit or network* or stakeholder* or support or outreach* or capacit* or mobili#ation or mobili#e or organi#ation or organi#e or inform* or educat* or instruct* or teach or empower* or train* or workshop* or leader* or service* or cent$3)).ti,ab,kw. (254235)
21. Community Health Services/or Community‐Institutional Relations/or Community Networks/or Community Health Centers/or Community Health Nursing/or Health Education/or Community Health Planning/or Community‐Based Participatory Research/or Community Participation/or Rural Communities/or Community Development/or Community Health Workers/or Community Health/or Community‐Based Distribution/or Capacity Building/or Health Care Delivery/or Mobile Health Units/or House Calls/or Patient Participation/or Patient Tracking/(373481)
22. 20 or 21 (568467)
23. 19 or 22 (8748532)
24. Parents/or Fathers/or Mothers/or Grandparents/or Caregivers/or Single Parent/or Pregnant Women/or Child, Preschool/or Infant/or Infant, Newborn/or Infant, Low Birth Weight/or Infant, Small for Gestational Age/or Infant, Very Low Birth Weight/or Infant, Extremely Low Birth Weight/or Infant, Postmature/or Infant, Premature/or Infant, Extremely Premature/(1555518)
25. (child* or infant* or newborn* or neonat* or neo nat* or prenatal or pre natal or antenatal or antenatal or baby or babies or toddler* or preschool* or parent* or mother* or father* or maternal or paternal).ti,ab,kw. (2260892)
26. 24 or 25 (2843161)
27. 16 and 23 and 26 (16661)
28. (random* or experiment* or (match* adj2 (propensity or coarsened or covariate)) or "propensity score" or ("difference in difference*" or "difference‐in‐difference*" or "differences in difference*" or "differences‐in‐difference*" or "double difference*") or ("quasi‐experimental" or "quasi experimental" or "quasi‐experiment" or "quasi experiment") or ((estimator or counterfactual) and evaluation*) or "instrumental variable*" or (IV adj2 (estimation or approach)) or regression discontinuity or time series or segment* regression).ti,ab,kw. (2935587)
29. Randomised Controlled Trial/or Random Allocation/or Evaluation Studies/or Propensity Score/or Interrupted Time Series Analysis/or Controlled Before‐After Studies/or Controlled Clinical Trial/or Non‐Randomised Controlled Trials as Topic/(885938)
30. 28 or 29 (3363578)
31. Cost Analysis/or Cost‐Benefit Analysis/or Quality‐Adjusted Life Years/or Economics, Medical/or Cost of Illness/or Health Care Costs/or Direct Service Costs/or Budgets/or Health Care Sector/or Public Expenditures/(191103)
32. (cost‐effective* or cost‐benefit).ti,ab,kw. (128028)
33. ("life year" or "life years" or qaly* or daly*).ti,ab,kw. (18238)
34. ((economic* or cost*) adj6 (mortality or death* or markov)).ti,ab,kw. (16865)
35. ("cost minimi*" or "cost‐utilit*" or "economic evaluation*" or "economic review*" or "cost outcome" or "cost analys*" or "economic analys*" or "budget* impact analys*").ti,ab,kw. (26939)
36. 31 or 32 or 33 or 34 or 35 (296674)
37. (review or meta‐analysis).pt. (2527031)
38. cochrane database of systematic reviews.jn. (14038)
39. (systematic review or literature review).ti. (126004)
40. 37 or 38 or 39 (2555693)
41. 30 or 36 or 40 (5870802)
42. 27 and 41 (5118)
43. exp Animals/(22120081)
44. Humans/(17568637)
45. 43 not (43 and 44) (4551444)
46. 42 not 45 (4978)

## APPENDIX 2 CRITICAL APPRAISAL OF QUALITATIVE AND DESCRIPTIVE QUANTITATIVE STUDIES INCLUDED TO ANSWER RESEARCH QUESTIONS 3 AND 4 ^8^ The appraisal tool is an adapted version of CASP (2018) and draws on Snilstveit et al.'s (2015) critical appraisal list.

### Critical appraisal of qualitative and descriptive quantitative studies

1. Is the research aim clearly stated? (Yes/No)

#### REPORTING

2. Description of the context? (Yes/No)
3. Description of sampling procedures? (Yes/No)
4. Are sample characteristics sufficiently reported? (sample size, location, and at least one additional characteristic) (Yes/No)
5. Is it clear how the data were collected (eg: for interviews, is there an indication of how interviews were conducted? (Yes/No)
6. Methods of recording of data reported? (Yes/No)
7. Methods of analysis explicitly stated? (Yes/No)

#### METHODOLOGY

8. Is there a clear link to relevant literature/theoretical framework? (Yes/No)
9. Is the design appropriate to answer the research question? (Yes/No)
10. Was the sampling strategy appropriate to the aims of the research? (Yes/No)
11. Were the data collected in a way that addressed the research issue? (Yes/No)
12. Was the data analysis sufficiently rigorous? (Yes/No)
13. Has triangulation been applied? (Yes/No)
14. Is the analysis and conclusions clearly presented? (Yes/No)
15. Does the paper discuss ethical considerations related to the research? (Yes/No)
